# Macula densa cell angiogenic mechanisms and their therapeutic potential in kidney disease

**DOI:** 10.3389/fbioe.2025.1606230

**Published:** 2025-08-06

**Authors:** Georgina Gyarmati, Dorinne Desposito, Anne Riquier-Brison, Alejandra Becerra Calderon, Greta Trogen, Audrey Izuhara, Michifumi Yamashita, János Peti-Peterdi

**Affiliations:** ^1^ Department of Physiology and Neuroscience, Zilkha Neurogenetic Institute, University of Southern California, Los Angeles, CA, United States; ^2^ Department of Pathology and Laboratory Medicine, Cedars-Sinai Medical Center, Los Angeles, CA, United States; ^3^ Department of Medicine, University of Southern California, Los Angeles, CA, United States

**Keywords:** progenitor cells, glomerular endothelium, extracellular matrix, angiogenesis, CCN1, lupus nephritis

## Abstract

**Introduction:**

The neuronally differentiated key sensory and regulatory cells of the macula densa (MD) were recently identified to control resident progenitor cells and endogenous kidney tissue remodeling and regeneration. Among the many MD-derived secreted angiogenic, growth, and extracellular matrix remodeling factors, cell communication network 1 (CCN1) is a known positive modulator of angiogenesis. This study aimed to validate and characterize the angiogenic potential and mechanisms of MD-derived biologicals including CCN1 in control healthy and disease conditions.

**Methods:**

The angiogenic effects of secreted MD cell factors including CCN1 on glomerular endothelial cells (GEnC) were studied in vivo using Cdh5-Confetti mice with endothelium-specific expression of multicolor genetic reporters for genetic cell fate tracking, and in vitro using cultured GEnCs in angiogenesis, cell proliferation and migration assays. The effects of treatment with conditioned MD cell culture media (rich in CCN1) were tested in NZM.2328 BAFF transgenic lupus mice using intravital multiphoton microscopy, histology, and classic kidney function phenotyping.

**Results:**

High CCN1 expression in MD cells was confirmed in the mouse and human kidney with diminished levels in patients with lupus nephritis. GEnC angiogenesis assays confirmed the strong angiogenic potential of CCN1 and the mechanistic role of integrins β3 and β5, VEGFR2 and Akt signaling in this process. Treatment of NZM.2328 BAFF transgenic lupus mice with MD factors vs control improved GEnC function, halted the progression of albuminuria, and reduced immune cell homing. The implantation of cultured MDgeo cells but not control M1 cells under the renal capsule of Cdh5-Confetti mice induced the growth of new blood vessels radially towards the MD cell epicenter that were formed by clonal expansion of host endothelial cells.

**Discussion:**

The present study confirmed the strong angiogenic effect of MD-derived factors including CCN1 that may be targeted in future biologicals, biofabricated tissue, or cell-based therapeutic developments for kidney diseases.

## Introduction

Chronic kidney disease (CKD) is one of the fastest growing health issues globally despite recent efforts and advances in (re)building or bioengineering functional kidney tissues for transplantation or in enhancing endogenous kidney tissue regeneration and repair (Francis et al., 2024; Little and Humphreys, 2022). The majority of the 850 million CKD patients worldwide have remaining functional kidney tissues with residing progenitor cells (Bussolati et al., 2005; Biancone and Camussi, 2014; Romagnani et al., 2013). One of the therapeutic strategies is to stimulate resident progenitor cells and their intrinsic mechanisms of tissue regeneration with the goal to achieve structural and functional regression of pre-existing kidney damage and CKD. Improved mechanistic understanding of endogenous renal tissue remodeling and regeneration, and identification of the most powerful cell and molecular targets are key to develop highly efficient, specific, and mechanism-based therapies.

Recently, our group discovered a key, but formerly overlooked, regulatory role of macula densa (MD) cells in glomerular, vascular, and interstitial tissue remodeling and regeneration (Gyarmati et al., 2024a). Unexpectedly, the roles include the synthesis and secretion of newly identified MD-specific angiogenic, tissue growth, patterning, and extracellular matrix (ECM) remodeling factors and their paracrine mechanistic actions to control resident progenitor cells and regulate endogenous kidney tissue remodeling and regeneration (Gyarmati et al., 2024a). One of the highly expressed and secreted MD-specific tissue remodeling factors is CCN1 (a.k.a. cysteine-rich protein, Cyr61), a member of the cell communication network (CCN) family of secreted matricellular proteins that also includes nephroblastoma overexpressed (NOV, CCN3) (Chen and Lau, 2009; Jun and Lau, 2011; Mo et al., 2002; Perbal, 2001a; Yang and Lau, 1991; Brigstock et al., 2003). CCN1 is an extracellular matrix (ECM)-associated angiogenic inducer, and is known to function as a physiological regulator of insulin-like growth factor (IGF1) and vascular endothelial growth factor (VEGF) signaling efficiency, and ultimately the adhesion, migration, proliferation, differentiation, and survival of vascular endothelial cells (Perbal, 2001a; Yang and Lau, 1991). CCN proteins are organized into four conserved modular domains including IGF binding peptide, von Willebrand factor type C repeat, thrombospondin type 1 repeat, and cysteine knot domains that reflect their functions as molecular crosslinkers of IGF1R and VEGFR2 receptors, integrins (ITG), and various ECM components to promote, transduce, and modulate growth factor and hormone signaling and sensing the extracellular environment (Chen and Lau, 2009; Jun and Lau, 2011). CCN1 promotes cell spreading and adhesive signaling, the expression of genes involved in angiogenesis and matrix remodeling, including VEGF-A, VEGF-C, type I collagen, matrix metalloproteinase 1 (MMP1), and MMP3 (Chen and Lau, 2009; Mo et al., 2002). CCN1 binds to target cells based on their specific integrin profile and the proteolytic cleavage of C or N-terminal domains is involved in the many versatile CCN1 functions and activities in various cell types, for example pro-angiogenic (in endothelial cells) and anti-fibrotic (in fibroblasts) effects (Chen and Lau, 2009; Jun and Lau, 2011; Mo et al., 2002; Perbal, 2001a; Yang and Lau, 1991; Brigstock et al., 2003; Perbal, 2001b). CCN1 can also bind to heparan sulfate proteoglycans (HSPGs) including syndecan (Chen et al., 2007). The many functions of CCN1 highly correspond and fit with the current knowledge and molecular biological details of glomerular cell and matrix biology (Chen et al., 2010; Lee, 2012; Kuro-o, 2010; Maarouf et al., 2016; Bach and Hale, 2015; Francis et al., 2016; Lausecker et al., 2022).

MD cells are strategically positioned at the glomerular entrance and are known to regulate renal and glomerular hemodynamics and renin release as their classic physiological functions (Peti-Peterdi, 2006; Peti-Peterdi and Harris, 2010; Peti-Peterdi et al., 2009). However, MD cells are also ideally positioned for the local, paracrine regulation of all glomerular and juxtaglomerular cell types including glomerular endothelial cells (GEnCs). Recently, our laboratory uncovered and visualized using intravital multiphoton microscopy (MPM) imaging the dense network of MD cell basal processes called maculapodia in MD-GFP transgenic mice (Gyarmati et al., 2021). The long MD processes densely arborize into the glomerular mesangium and around the glomerular afferent/efferent arterioles and contain secretory vesicles with a diverse cargo of angiogenic and tissue remodeling factors including Pappalysin 2 (PAPPA2) and CCN1 (Gyarmati et al., 2024a; Gyarmati et al., 2021). The diffusion of MD-derived factors into the glomerular mesangium, capillary plasma, and the renal interstitium is further propelled by the bulk interstitial fluid flow in the juxtaglomerular apparatus (JGA) area that was visually demonstrated in the intact living kidney using MPM imaging (Rosivall et al., 2006). MD cells feature a high rate of protein synthesis regulated by mTOR signaling (Shroff et al., 2021). Low dietary salt intake, MD mTOR and Wnt signaling upregulate the synthesis of both traditional and novel MD proteins including PAPPA2 and CCN1 (Gyarmati et al., 2024a; Shroff et al., 2021). Single-cell genetic cell fate tracking in Cdh5-Confetti mice with serial intravital MPM imaging confirmed clonal GEnC remodeling by local rather than circulating endothelial precursor cells (EPCs) localized at the glomerular vascular pole and in the glomerular arterioles closest to the MD (Desposito et al., 2021).

The newly identified tissue remodeling functions of MD cells strongly suggest the potent angiogenic effect of MD-derived factors including CCN1. This study aimed to validate and characterize the angiogenic potential and mechanisms of MD-derived biologicals including CCN1 in control healthy and kidney disease conditions. The condition of lupus nephritis (LN) was chosen as the disease model, because the diverse pro-angiogenic, anti-fibrotic, and anti-inflammatory functions of CCN1 (Kerek et al., 2025) are relevant to the complex vascular, fibrotic, and immune pathology of LN. The ultimate goal of these efforts is to develop powerful tissue regenerative therapies for CKD and its cardiovascular complications by enhancing endogenous kidney tissue regeneration.

## Materials and methods

### Animals

Male and female, 6–8 weeks old C57BL6/J mice (Jackson Laboratory, Bar Harbor, ME) or 8–12-months-old NZM.2328 lupus nephritis (LN) mice were used in all experiments. Inducible and conditional homozygous Cdh5-Confetti fluorescent reporter mice on the C57BL6/J background, which specifically express multicolor fluorescent reporter proteins (membrane targeted CFP, nuclear GFP, cytosolic YFP and RFP) in vascular endothelial cells were generated by crossing mice expressing Cre-ERT2 recombinase under the control of the Cdh5 promoter (originally developed by Ralf Adams, Cancer Research UK Scientist, and obtained from Cancer Research Technology Limited) (Wang et al., 2010) and mice with the R26 R-Confetti construct (The Jackson Laboratory) (Snippert et al., 2010) as described before (Gyarmati et al., 2024a). Tamoxifen was administered 75 mg/kg by oral gavage for a total of three times (every other day) for full induction in appropriate animal models. The NZM.2328 recombinant inbred mouse strain was used as a mouse model of lupus nephritis (Kadoya et al., 2020; Rudofsky and Lawrence, 1999). NZM.2328 mice have been extensively characterized previously and are known to develop severe proteinuria (>300 mg/dL) and acute glomerulonephritis by 12 months of age (Waters et al., 2001). Mice with urine albumin levels over 300 mg/mL on at least two sequential urine tests were included in this study.

After 2 weeks of washout period a subset of the Cdh5-Confetti animals received a single dose of renal subcapsular VEGF (0.25 µg/injection) (n = 7), CCN1 (2 µg/injection, PeproTech/Thermo Fisher) (n = 10), or renal subcapsular MDgeo or M1 cell implantation (1–5 µL/injection 100K cell/mL). NZM.2328 LN mice received chronic daily intraperitoneal (ip) injections of either low salt conditioned MD cell media (200 µL/day) or isovolumetric vehicle (DMEM-F12, Thermo Fisher) treatment over 4 weeks (n = 8 each). All animal protocols were approved by the Institutional Animal Care and Use Committee at the University of Southern California.

### Serial *in vivo* multiphoton microscopy (MPM)

Under continuous anesthesia (isoflurane 1%–2% inhalant via nose cone), the animals were placed on the stage of the inverted microscope as described previously (Gyarmati et al., 2024a; Desposito et al., 2021; Hackl et al., 2013). Body temperature was maintained with a homeothermic blanket system (Harvard Apparatus). Alexa Fluor 680–conjugated bovine serum albumin (Invitrogen, Thermo Fisher) was injected iv to label the plasma. The images were acquired using a Leica SP8 DIVE multiphoton confocal fluorescence imaging system with a ×40 Leica water-immersion objective (numerical aperture 1.1) powered by a Chameleon Discovery laser at 960 nm (Coherent) and a DMI8 inverted microscope’s external Leica 4Tune spectral hybrid detectors (emission at 510–530 nm for FITC/Alexa488 and 660–750 nm for AF680, and 475–485 nm for detecting second harmonic generation) (Leica Microsystems). The potential toxicity of laser excitation and fluorescence to the cells was minimized by using a low laser power and high scan speeds to keep total laser exposure as low as possible. The usual image acquisition (12-bit, 512 × 512 pixel) consisted of single *Z*-stack per tissue volume (<1 min), and 3–5 min time series (xyt, 526 ms per frame) with the Leica LAS X imaging software and using the same instrument settings (laser power, offset, gain of both detector channels) which resulted in no apparent cell injury. The strong, positive, cell-specific signal (immune cells, endothelial glycocalyx) and high-resolution MPM imaging allowed for easy identification of vascular/tubular structures and immune cells. Serial imaging of the same glomerulus in the same animal/kidney was performed over 4 weeks. The detailed method of serial survival imaging with surgical kidney exteriorization was described before (Gyarmati et al., 2024a; Desposito et al., 2021; Hackl et al., 2013).

### *In vivo* labeling

Alexa Fluor 488–labeled anti-CD44 antibodies (BioLegend) were used to detect activated memory T cells and directly and quantitatively visualize their glomerular intravascular homing in NZM.2328 mice using serial MPM. These antibodies were administered via retro-orbital sinus injections each at a dose of 30 μL as described previously (Kadoya et al., 2020; Gyarmati et al., 2022). Clearly positively labeled cells were seen immediately after antibody injection. The number of CD44 positive cells in entire glomeruli were calculated in 3D volume (*xyz*) images before and after treatment. Multiple glomeruli per each mouse were analyzed as indicated to avoid sampling errors. FITC-WGA lectin (*Triticum vulgaris*; L4895, MilliporeSigma), administered via retro-orbital sinus at 2 μg/g body weight, was used to visualize the glomerular endothelial surface layer (glycocalyx) (Kadoya et al., 2020; Gyarmati et al., 2022). FITC-WGA lectin fluorescence and thickness were evaluated before and after treatment. Quantification of glycocalyx thickness was performed on linear profiles by calculating the width of FITC-WGA signal at half-maximum fluorescence intensity as described before (Kadoya et al., 2020; Gyarmati et al., 2022; Butler et al., 2019; Salmon et al., 2012).

### Urine albumin to creatinine ratio (ACR)

Spot urine was collected from animals before and after treatment. Urine albumin was measured by using murine microalbuminuria ELISA kit (Albuwell M kits, Exocell). Urine creatinine was measured via microplate assay (The Creatinine Companion, Exocell), and ACR was calculated.

### Tissue processing, immunohistochemistry, and histology

After anesthesia with a combination of ketamine (100 mg per kg body weight) and xylazine (10 mg per kg body weight), animals were perfused with ice-cold PBS into the left ventricle followed by ice-cold 4% paraformaldehyde (PFA) for 2 min each, and tissues were fixed with 4% PFA at 4°C overnight. To visualize Confetti colors, tissues were embedded in OCT after sucrose cryoprotection method (30% sucrose at room temperature for 3 h) and flash frozen. Cryosections (25 μm thickness) were imaged using the same Leica TCS SP8 microscope as above. Confetti^+^ clonal or unicolor tracing units were defined as numerous directly adjacent individual cells that featured the same Confetti color combination. The methods and use of merged CFP/GFP/YFP/RFP images to identify all 10 possible Confetti color combinations were described previously (Desposito et al., 2021; Gyarmati et al., 2024b). The counting of Confetti^+^ cells and clones was facilitated by standardized image thresholding using ImageJ (NIH), LAS X software (Leica Microsystems Inc.), and cell-counting algorithms of Imaris 10.4 3D image visualization and analysis software (Bitplane) for imaging same-size *Z*-stacks (Gyarmati et al., 2024a; Desposito et al., 2021; Gyarmati et al., 2024b). Immunofluorescence staining was performed in paraffin embedded human kidney sections (8 μm thickness). After antigen retrieval (8 min at 95°C in citrate buffer using pressure cooker) and blocking (30 min in goat blocking buffer), the sections were incubated with anti-CCN1 (1:100; sc1311, Santa Cruz Biotechnology, Dallas, TX) primary antibodies followed by incubation with the secondary antibodies conjugated with Alexa 594 (1:500; A-11012, Invitrogen). Slides were mounted by using DAPI-containing mounting media (VectaShield, Vector Laboratories Inc).

### *In-vitro* angiogenesis, cell migration and cell proliferation assay, and immunoblotting

Glomerular endothelial cells (GEnC) from a tsA58 mouse cell line (Akis and Madaio, 2004) were grown and subcultured in DMEM‐based normal GEnC growth media as described previously (Becerra Calderon et al., 2024; Toma et al., 2008). GEnCs were exposed to overnight serum starvation before experiments. For the tube formation assay cells were removed from flasks using Trypsin/EDTA solution. After washing GEnCs were resuspended in non-supplemented GEnC media to ensure homogenous single cell suspension. Cells were diluted (4 × 10^4^ cells/200 µL) in non-supplemented media in the presence or absence of angiogenesis inducers and inhibitors (CCN1 0, 10, 100, 500 ng/mL, VEGF 80 ng/mL, CCN1+ anti-VEGFR2, CCN1+ anti-ITGB3, CCN1+ anti-ITGB5 antibodies, Abcam). Cells (4 × 10^4^/well in 200 µL media) were plated in gel-coated bottom glass 96 well plates. Cells were incubated at 37°C and 5% CO2. Tube formation was quantified by measuring the number of circular capillary-like structures (tubes) in two-dimensional microscope images after 12 and 24 h. For cell migration scratch assay GEnCs were cultured in 24 well plates. At 85% of confluence cells were starved overnight, GEnC cell culture media supplemented with angiogenesis inducers and inhibitors was added as described above. A straight scratch across the cell monolayer was created using a sterile pipette tip. Brightfield light microscope images of the scratch area were taken at baseline and every 6 h thereafter for 24 h. The wound closure was measured using ImageJ image analysis software area measurement tools. Scratch opening percentage was determined via the ratio of the area at 24 h over the area at baseline. Trypan Blue Cell Counting assay was performed to measure GEnC proliferation in response to angiogenesis inducers and inhibitors as detailed above. For GEnC proliferation assay, cells were removed and resuspended to ensure homogenous single cell suspension as described above. Cells were diluted (1 × 10^4^ cells/100 µL) in non-supplemented media in the presence or absence of angiogenesis inducers and inhibitors as above. 5 × 10^4^ cells were seeded in each well. After 24 h cells were removed, and viable cells were counted manually using Incyto C-Chip Disposable Hemacytometers (Grayline Medical Inc., Norwalk, CA). For immunoblotting, cultured GEnCs were homogenized in a buffer containing 20 mM Tris·HCl, 1 mM EGTA pH 7.0, and a protease inhibitor cocktail (BD Bioscience). Protein (20 μg) was processed for immunoblotting as described previously (Desposito et al., 2021). Primary antibodies and dilutions were anti-phospho (Ser473) and total protein kinase B antibodies (AKT, 1:1,000, CST4060, CST2920, Cell Signaling Technology, Danvers, MA), and anti-phospho (Y1054 + Y1059) and total VEGF Receptor 2 antibodies (VEGFR2, 1:1,000, ab5473, ab39638, Abcam, Cambridge, United Kingdom). After incubation, blots were incubated with secondary antibodies (1:5,000; LI-COR Biosciences) and then visualized with Odyssey Infrared Imaging System (LICOR Biosciences). Protein staining of the gel (Coomassie staining) was performed and analyzed to confirm equal loading as described previously (Desposito et al., 2021).

### MD^geo^ and M1 cell culture

Immortalized macula densa cell line (MD^geo^) and M1 cells (a mouse cortical collecting duct cell line obtained from the American Type Culture Collection, ATCC, Manassas, VA) were cultured in sterile conditions as described before (Gyarmati et al., 2024a; Kang et al., 2008). MD^geo^ cells were cultured at 33°C in DMEM-F12 cell culture media supplemented with nerve growth factor (NGF, 0.1 µg/mL; N8133, Millipore Sigma), 10% Fetal Bovine Serum, (FBS, Thermo Fisher, Waltham, MA), 1% Penicillin-Streptomycin (P/S 10,000 U/mL, Thermo Fisher) and 0.0005% of Dexamethasone. For differentiation, cells were incubated at 37°C, 5% CO_2_ for 14 days in complete MD cell culture media supplemented with NGF (0.1ug/mL; N8133, Millipore Sigma). Differentiated MD^geo^ cells or M1 cells were removed from flasks using TrypLE express enzyme (#12604021, Thermo Fisher) treatment, washed and resuspended in sterile saline solution (10^5^ cells/mL). Subcapsular implantation was performed by the injection of 1–2 µL solution (approximately 100–200 cells) in a single area of the left kidney.

### Generation of conditioned MD^geo^ cell culture media

After full differentiation, MD^geo^ cells were washed in PBS to remove FBS and NGF and were physiologically activated by temporary exposure to low-salt conditions (low salt (67 mM NaCl) vs. control [135 mM NaCl) DMEM-F12 medium, isosmotic with added mannitol (Yang et al., 2000)] for 6 h every other day (3 times). After final conditioning, the cell culture media was collected in sterile conditions and stored at −80 C until treatment.

### Human tissues

Histological sections of renal biopsies from patients with clinical and laboratory evidence of lupus nephritis were used from the Cedars-Sinai Medical Center’s biobank approved by IRB protocol Pro00046999. Healthy unaffected parts of renal tumor biopsies from patients served as control as reported (Gyarmati et al., 2024a).

### Statistics

Data represent average ± SEM and were analyzed using Student’s t tests (between two groups) or one-way ANOVA (for multiple groups) with *post hoc* comparison by Dunnett’s, Tukey’s, Šidák’s or Fisher’s tests as appropriate. P values of less than 0.05 were considered significant. Statistical analyses were performed using GraphPad Prism 9.0c.

## Results

### The angiogenic effects of CCN1 on glomerular endothelial cells

**FIGURE 1:**
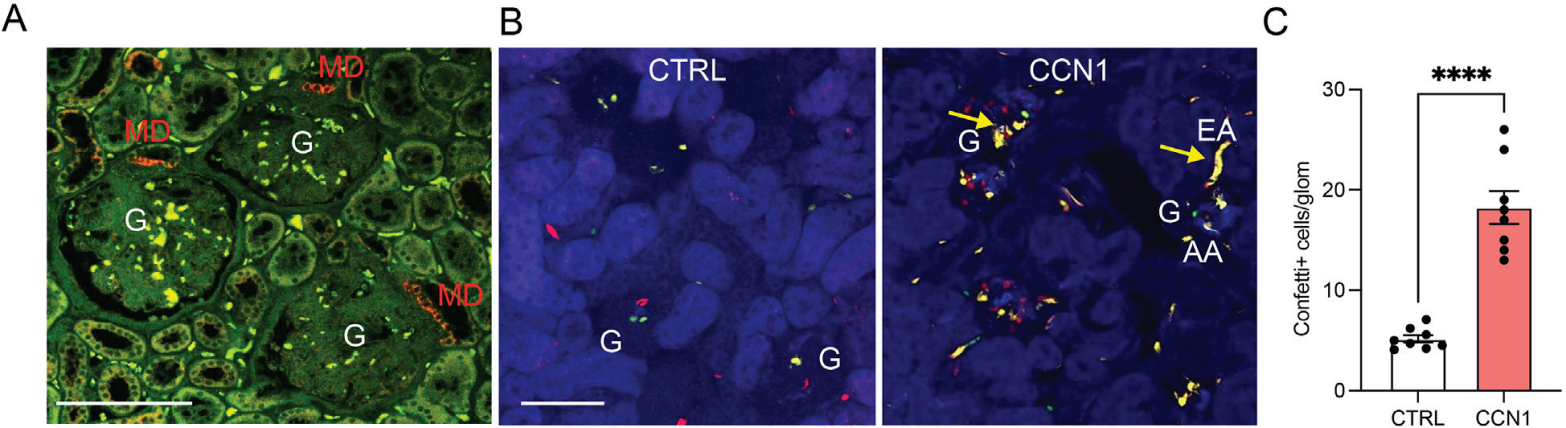
CCN1 expression and angiogenic effects *in vivo*. **(A)** Immunofluorescence localization of CCN1 expression (red) in the healthy human kidney. Tissue autofluorescence is shown in green. Note the exclusively macula densa (MD)-specific CCN1 expression in three adjacent nephrons. G, glomerulus, scale bars are 100 μm. **(B)** Native fluorescence images of fixed frozen kidney tissue sections from control (left) and CCN1-treated (right) Cdh5-Confetti mice, in which endothelial cells are genetically labeled by the expression of either membrane-targeted CFP (blue), nuclear GFP (green), cytosolic YFP (yellow) or cytosolic RFP (red). Note the low number of Confetti+ single peritubular and glomerular endothelial cells (GEnCs) in control (left), but their high density in response to single dose subcapsular CCN1 treatment (2 µg/injection) (right). Arrows show yellow unicolor (clonal) afferent (AA) and efferent arteriole (EA) GEnC progenies at the glomerular (G) vascular poles in response to CCN1 treatment (right). **(C)** Summary of the number of Confetti+ cells per glomerulus in control and CCN1-treated mice. Data represent mean ± SEM. **** p < 0.0001, n = 8 mice/group, using t-test.

To visually demonstrate the presence and mechanisms of MD cell-mediated angiogenesis, we first performed immunolocalization of the angiogenic inducer and modulator CCN1 in the healthy human kidney. Consistent with a recent report that identified MD-specific CCN1 expression in the mouse and human kidney at the RNA and protein level (Gyarmati et al., 2024a), CCN1 immunofluorescence labeling was found exclusively in MD cells in all nephrons (Figure 1A).

Subsequent *in vivo* studies used Cdh5-Confetti mice with inducible Cre/lox mediated expression of the multicolor Confetti reporter specifically in vascular endothelial cells. This powerful *in vivo* model was established recently for the genetic cell fate tracking of vascular endothelial cells at the single-cell level (Desposito et al., 2021; Manavski et al., 2018). After 2 weeks of tamoxifen washout, mice were treated with single injections of either vehicle (PBS) or recombinant CCN1 (2 µg/injection) under the renal capsule of the left kidney. Mice were followed for 1 week and then processed for tissue harvest and histological analysis. Quantitative imaging of frozen kidney sections confirmed the strong angiogenic effects of CCN1 *in vivo*. As illustrated in Figure 1B, a few Confetti+ cells appeared in glomeruli in a stochastic distribution in control, but the number of Confetti+ cells increased considerably in response to CCN1 treatment for 1 week suggesting GEnC proliferation (Figure 1C). In addition, unicolor (clonal) GEnC progenies appeared at the glomerular vascular poles in multiple nephrons including in the terminal segments of the afferent/efferent arterioles (Figure 1B), suggesting the presence of highly active single EPC-derived endothelial remodeling and angiogenesis. The observed 4-fold increase in the number of Confetti+ GEnCs in response to CCN1 was similar in extent to the effect of VEGF as reported recently (Desposito et al., 2021).

**FIGURE 2:**
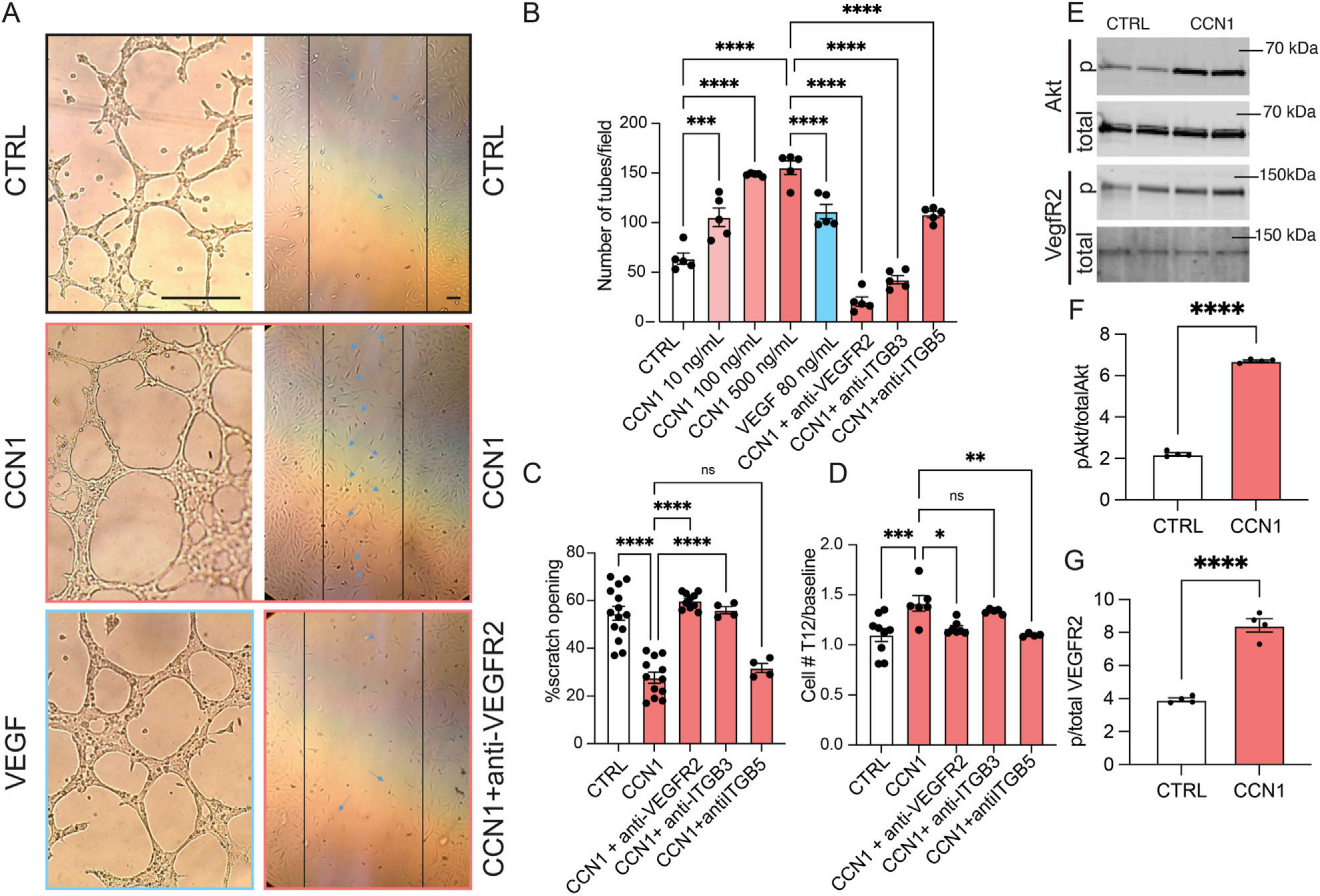
The stimulatory effects and signaling mechanisms of CCN1 on GEnC angiogenesis, migration, and proliferation *in vitro*. **(A)** Brightfield images of GEnCs cultured in control cell medium or supplemented with CCN1 (100 ng/mL) and VEGF (80 ng/mL) (left column) and in the scratch assay with control, CCN1 (100 ng/mL), and anti-VEGFR2 (2 μL/mL) treatment (right column). Note the reduced width of scratch opening (indicated by vertical lines) in response to CCN1 treatment. Scale bars are 25 μm. **(B–D)** Statistical summary of angiogenesis (tube formation assay, B), cell migration (scratch assay, C), and proliferation assays (D) of GEnCs cultured *in vitro* with CCN1 (100 ng/mL) with or without blocking VEGFR2, anti-integrin β3, or β5 antibodies (2 μL/mL each). **(E–G)** Immunoblotting studies of total and phospho-Akt and VEGFR2 in GEnCs cultured in control or CCN1 (100 ng/mL) containing culture medium (n = 4 each). Data represent mean ± SEM, ns: not significant, *,**,***,**** p < 0.05–0.0001, using t-test or one-way ANOVA followed by Sidak’s test.

To better understand the molecular mechanisms of MD CCN1-mediated glomerular angiogenesis, cultured GEnCs were treated with vehicle control or CCN1 in conjunction with classic *in vitro* angiogenesis, cell proliferation and migration assays. Exogenous CCN1 added to the media of cultured GEnCs markedly stimulated angiogenesis in a dose-dependent manner (Figures 2A,B). CCN1 appeared to be an equally effective inducer of GEnC tube formation compared to the gold-standard VEGF (Figures 2A,B). CCN1 had similar robust positive effects on GEnC proliferation and cell migration (scratch opening assay) (Figures 2A–D). The pro-angiogenic effects of CCN1 were almost completely abolished in the presence of blocking antibodies against integrins α_v_β_3_ (for angiogenesis and migration) and α_v_β_5_ (for proliferation) and VEGFR2, suggesting that CCN1 is an important amplifier of VEGF and extracellular matrix (ECM) signaling to enhance GEnC proliferation, migration, and angiogenesis. Additional GEnC cell culture experiments identified the involvement of Akt and VEGFR2 as downstream signaling mechanisms of CCN1-induced angiogenesis (Figures 2E–G).

### Treatment with MD biologicals improves endothelial and kidney functional repair in lupus nephritis

**FIGURE 3:**
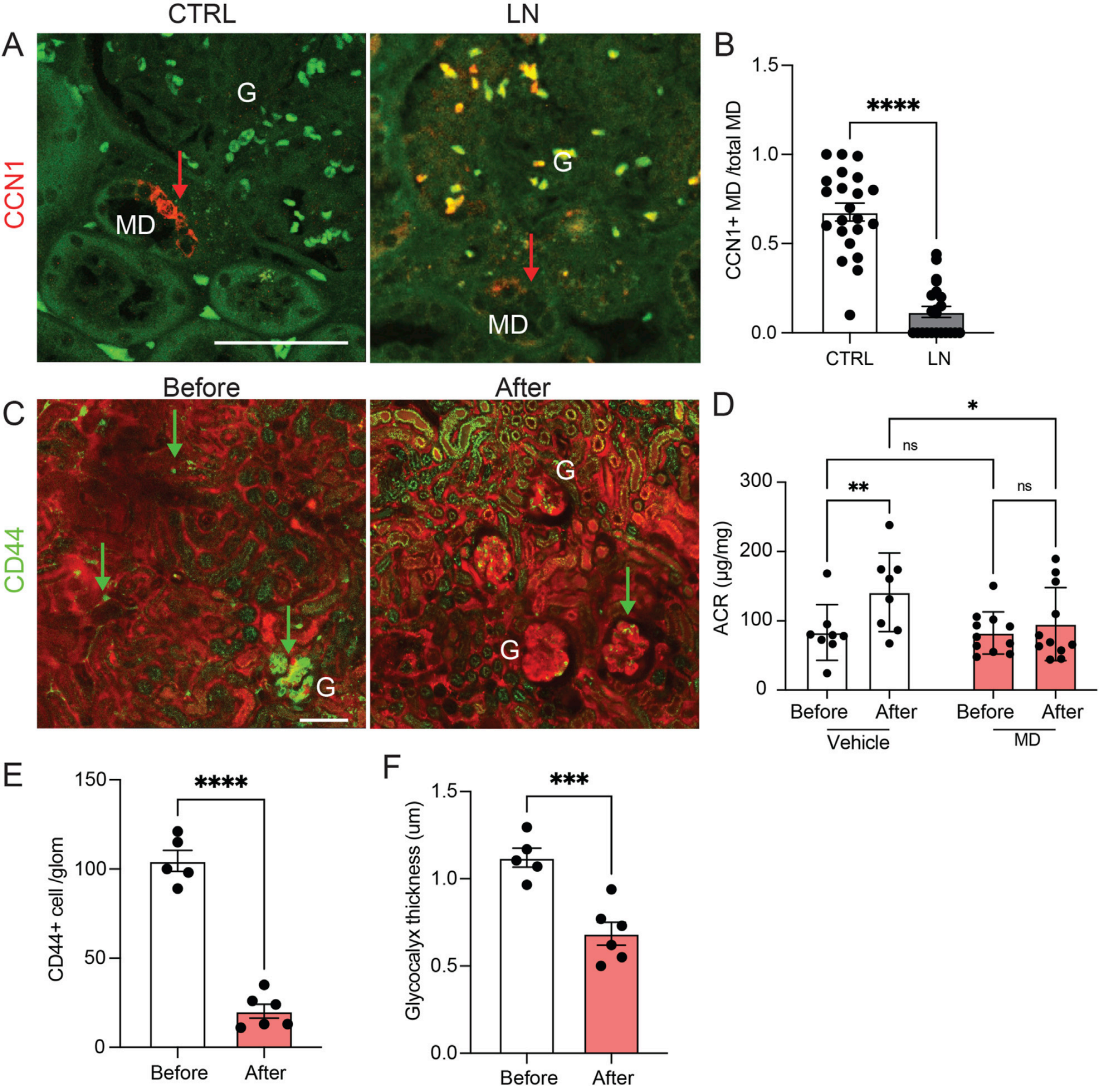
The renal expression and therapeutic effect of the angiogenesis modulator CCN1 in human and mouse lupus. **(A,B)** CCN1 immunofluorescence (red, arrows) images of human kidney tissue sections from control (CTRL) and lupus nephritis (LN) patient biopsies (A), and quantification of the ratio of CCN1+ vs all macula densa (MD) cells in a juxtaglomerular area (B). Note the strong CCN1 expression exclusively in cells of the MD in controls and mostly absent labeling in kidney tissue samples from patients with LN. Green is tissue autofluorescence, (G), glomerulus, n = 8 each (average of 5 MDs/sample), ****P < 0.0001 using t-test. Scale bars are 50 μm. **(C–F)** The renoprotective effects of treatment with MD biologicals (conditioned MDgeo cell culture media) for 4 weeks in the NZM.2328 BAFF TG mouse model of LN. **(C)** Serial *in vivo* multiphoton microscopy images of the renal cortex of NZM.2328 BAFF TG mice before and after 4 weeks of treatment with MD biologicals. Plasma was labeled by iv injected albumin-Alexa Fluor 594 (red). Note the high number of CD44^+^ activated memory T cells (green, arrows, labeled by iv injected anti-CD44-Alexa Fluor 488 antibodies) and their preferential glomerular vs. peritubular capillary homing before treatment. Tubular tissue autofluorescence is visible (green). **(D)** Changes in albuminuria (albumin/creatinine ratio, ACR) followed in the same mice before and after 4 weeks of treatment with either vehicle (DMEM-F12 control culture medium) or MD biologicals. Note the significant progression of albuminuria in the vehicle control vs MD treatment groups. **(E,F)** Quantification of the number of CD44^+^ immune cells/glomerulus (E) and the thickness of the endothelial glycocalyx labeled by FITC-WGA lectin (F) before and after 4 weeks of treatment with MD biologicals. Data represent mean ± SEM, n = 5–6 (multiple glomeruli/sample), ns: not significant, *,**,***,**** p < 0.05–0.0001 using t-test or two-way ANOVA with Fisher’s test.

To study the relevance of the MD angiogenic and tissue remodeling mechanisms in the human condition and human kidney disease, we first analyzed the expression of the angiogenic factor CCN1 in freshly nephrectomized and fixed human kidney tissues from patients with normal kidney function or with clinical and laboratory evidence of lupus nephritis (LN). CCN1 protein expression was entirely MD specific and CCN1 immunolabeling was present in almost all MD cells in normal human renal tissues (Figure 3A). However, the number of CCN1+ MD cells was significantly reduced to almost undetectable levels in LN, while the total MD cell number appeared unaltered based on the characteristic histological features of the densely populated tubular cell segment of the MD (Figures 3A,B). Recent studies confirmed that CCN1 expression is reduced in human LN and that MD cell number was maintained in CKD based on immunolabeling of the classic MD markers NOS1 and COX2 (Gyarmati et al., 2024a).

The endothelial and renoprotective effects of MD-derived biologicals were tested in the robust, human-like lupus nephritis model of NZM.2328 BAFF transgenic mice (Kadoya et al., 2020) (Figures 3C–F). Chronic treatment of preexisting LN was initiated using daily ip. injections of either control DMEM-F12 culture medium or low salt-conditioned cell culture medium of the MD^geo^ cell line as established and described recently (Gyarmati et al., 2024a), and animals were followed up for 4 weeks of treatment (Figures 3C–F). Albuminuria (ACR) was significantly less in LN mice treated with conditioned MD media compared to LN mice treated with control media (Figure 3D). Quantitative serial intravital MPM imaging performed at the baseline before treatment confirmed the substantial renal homing of CD44^+^ activated memory T cells preferentially in glomeruli (Figure 3C left, E) and the LN characteristic heterogenous accumulation of GEnC glycocalyx (Kadoya et al., 2020) (Figure 3F). In contrast, CD44^+^ cell number and GEnC glycocalyx thickness significantly reduced and improved by treatment with MD biologicals (Figure 3C, right, E,F). Recent studies confirmed the high CCN1concentration of conditioned MD media (Gyarmati et al., 2024a).

### The angiogenic effects of MD cells implanted under the renal capsule *in vivo*

**FIGURE 4:**
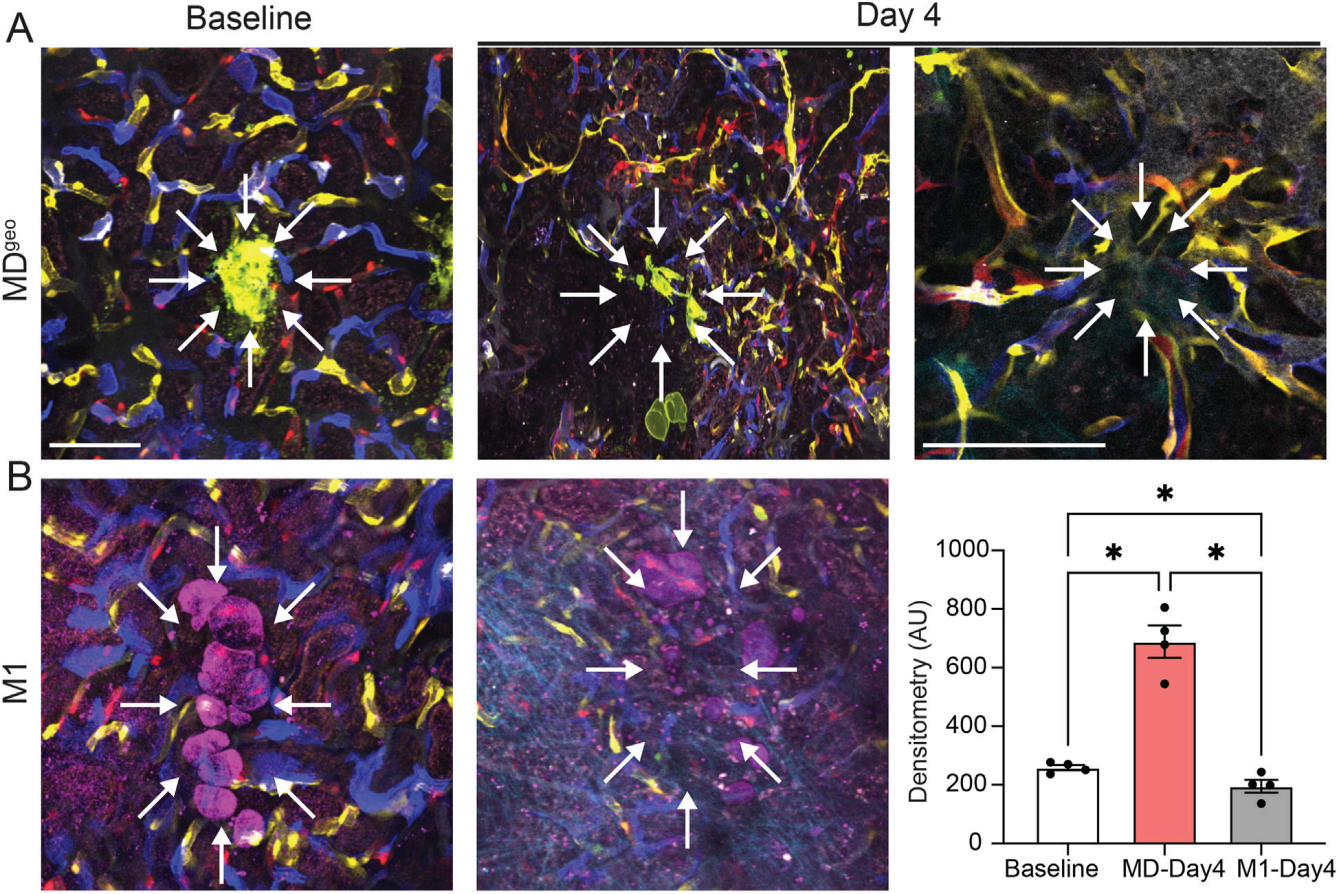
*In vivo* validation of MD cell-mediated angiogenesis. **(A,B)** Serial intravital MPM image pairs (A, B, left and center) of the renal subcapsular space in Cdh5-Confetti mice, in which single endothelial cells are specifically labeled and identified in one of four Confetti colors (CFP/GFP/YFP/RFP). Baseline (A, B, left) represents the time immediately following the implantation of either the recently developed MD cell line MD^Geo^ (A), or control M1 collecting duct cells (D) under the renal capsule of Cdh5-Confetti mice. Note the random distribution of multi-color peritubular capillaries. MD^Geo^ cells are identified based on their expression of membrane-targeted GFP (A, green), while M1 cells are shown by THG signal (B, magenta). Serial MPM images of the same renal cortical area 4 days after the implantation of MD^Geo^ (A, center) or M1 cells (B, center). Note the robust endothelial sprouting, neovascularization (angiogenesis) in the sub-capsular space around MD cells (A, center, right), but not around control M1 cells (B, center). High magnification *in vivo* MPM image demonstrating the radial pattern of new vessel growth towards the implanted MD cells’ epicenter (arrows) that contain circulating red blood cells (A, right). Most of the new capillaries appear in unicolor (identical Confetti color). Scale bars are 25 μm. Statistical summary of Confetti+ pixel density per area (B, right). Data represent mean ± SEM, n = 4 each, *P < 0.05 using t-test.

To validate the angiogenic effects of MD cells *in vivo*, cultured and low-salt conditioned MD cells were implanted under the renal capsule of Cdh5-Confetti host mice (Figure 4). *In vivo* MPM imaging of the implant area was performed serially for multiple days, starting in less than 30 min following the implantation of MD^geo^ cell clusters. In contrast to the random distribution of multi-color (non-clonal) Confetti+ capillaries of the host renal tissue at baseline, MPM imaging of the same implant areas on day 4 found robust endothelial sprouting, neovascularization (angiogenesis) around MD^geo^ cells (Figure 4A). High magnification MPM imaging revealed the growth of numerous new vessels towards the implanted MD cell group epicenter in a radial (star-shaped) pattern that contained circulating red blood cells (Figure 4A, right). Most of the new Confetti+ capillaries appeared in unicolor (clonal), suggesting they were formed by locally expanding EPCs (Figure 4A). A lack of evidence of endothelial cell sprouting or neovascularization was found 4 days following subcapsular implantation of M1 cells *in vivo*, confirming specificity of MD cell secretion of angiogenic factors (Figure 4B).

## Discussion

Here we performed a comprehensive imaging-based study to visually demonstrate and confirm the therapeutically relevant, strong angiogenic potential and mechanisms of MD cells and MD-derived biologicals. We report that CCN1, one of the predominant secreted angiogenic factors found in many organ tissues within the body, is specifically expressed by MD cells in the human kidney. Genetic cell fate tracking of GEnCs *in vivo* in control healthy mice revealed CCN1-induced GEnC proliferation and clonal vascular remodeling by resident endothelial progenitor cells at the glomerular vascular pole. *In vitro* GEnC cell culture studies identified the important mediator role of β3 and β5 integrins, VEGFR2 and Akt signaling in CCN1-induced angiogenesis. The angiogenic and tissue remodeling actions of MD biologicals translated to renoprotective therapeutic effects in a preclinical *in vivo* lupus nephritis model of chronic kidney disease. MD cell implantation under the renal capsule in mice induced star-shaped *de novo* angiogenesis, suggesting the release of angiogenic, cell growth and patterning factors from MD cells, and their powerful tissue remodeling effects *in vivo*. These insights strongly suggest that MD-derived biologicals including CCN1 are centrally involved in glomerular vascular biology and disease pathobiology and have therapeutic potential in CKD.

**FIGURE 5:**
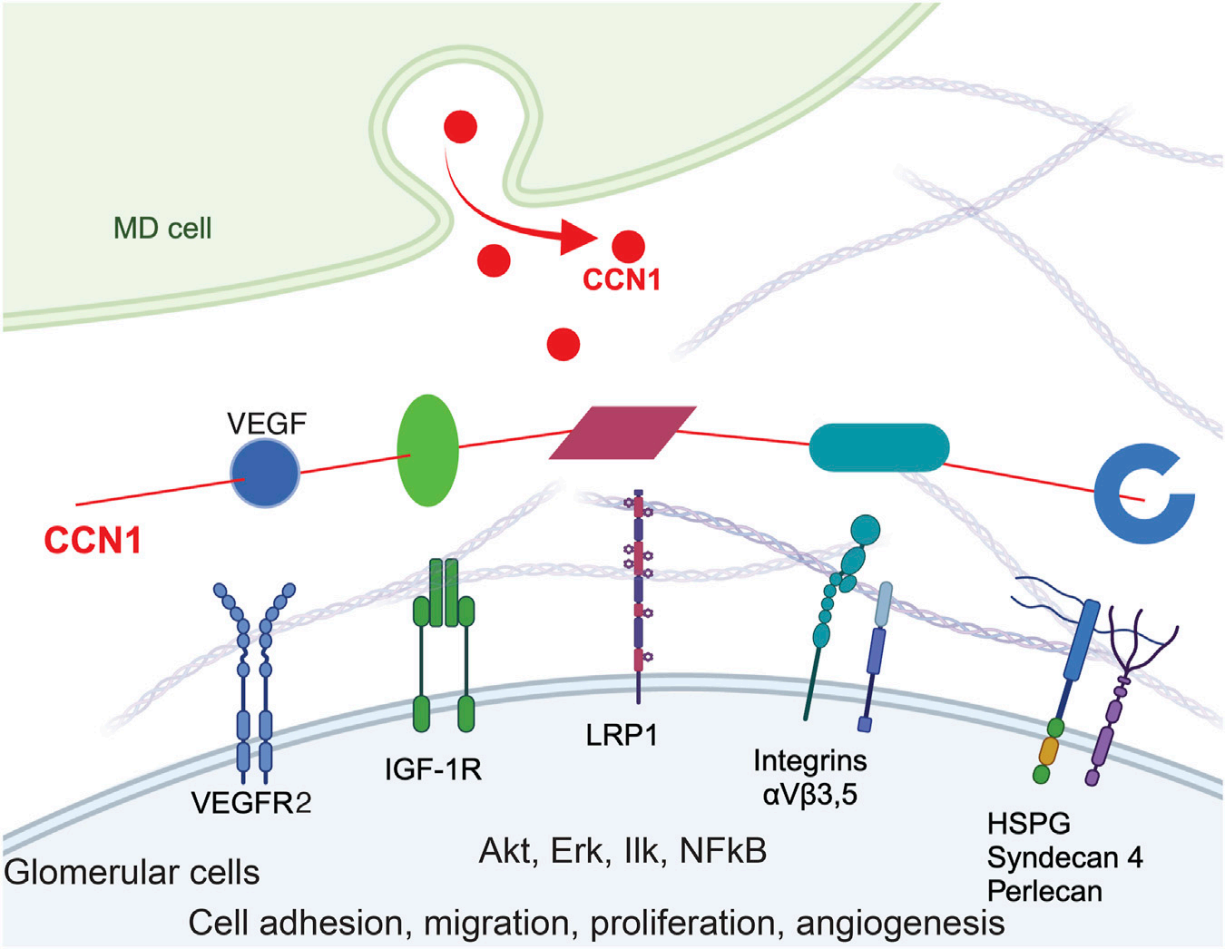
Model of CCN1 signaling in MD-to-GEnC crosstalk that regulates glomerular endothelial cell maintenance and regeneration. CCN1 secreted from MD cells acts as a physiological regulator and crosslinker of VEGFR2/ITG/ECM to augment VEGF signaling efficiency, and ultimately angiogenesis, GEnC proliferation and cell survival. The four main molecular domains of CCN1 are illustrated, including the IGFBP domain (green), a von Willebrand type C domain (vWC, magenta), a thrombospondin-1 domain (TSP-1, cyan) and a cysteine knot (blue). CCN1 also binds to VEGFR2 (blue) and augments VEGF signaling.

The present study is a logical continuation of the recent discovery of a novel endogenous kidney tissue remodeling and regeneration program orchestrated by cells of the MD under physiological activation conditions (Gyarmati et al., 2024a). This new MD function involves the synthesis and secretion of a specific set of angiogenic, tissue growth, patterning, and ECM remodeling factors and their paracrine actions to control resident progenitor cells and regulate kidney tissue remodeling (Gyarmati et al., 2024a). Here we explored the angiogenic and tissue remodeling potential of one such MD factor (CCN1, both *in vivo* and *in vitro*, Figures 1, 2) as well as all MD factors combined (MD cells and MD-secreted biologicals *in vivo*, Figures 3, 4). CCN1 was recently identified as a top highly expressed and secreted MD-specific factor (Gyarmati et al., 2024a). The presently observed CCN1-induced angiogenic effects are consistent with the previously reported CCN1 functions and activities in various cell types, for example pro-angiogenic (in endothelial cells) and anti-fibrotic (in fibroblasts) effects (Chen and Lau, 2009; Jun and Lau, 2011; Mo et al., 2002; Perbal, 2001a; Yang and Lau, 1991; Brigstock et al., 2003; Perbal, 2001b). Tissue protective therapeutic effects of CCN1 have been reported for many organs but mostly in the skin and liver (Kim et al., 2018), where CCN1 induced anti-fibrotic and cell proliferative responses by endothelial as well as other cell types (Kim et al., 2015). The four molecular domains of CCN1 (illustrated in Figure 5) explain well its physiological regulator function to control angiogenesis and tissue remodeling (Kim et al., 2018). These include domains that bind to growth factors, integrins, fibronectin, HSPG, and other ECM components to regulate target cell proliferation, adhesion, migration, and differentiation (Figure 5). Our results identified the integrins β3 and β5 as mediators of CCN1-induced GEnC angiogenesis, proliferation, and migration (Figure 2), which is consistent with the mechanisms reported for endothelial cells in other organs throughout the body (Kim et al., 2018). It should be noted that CCN1is known as an immediate-early response gene, which can explain the earlier reports on the acute and temporary expression of CCN1 in select tubular cells and podocytes, including in kidney injury (Muramatsu et al., 2002; Sawai et al., 2007). In contrast, MD cells constitutively express high levels of CCN1 (Gyarmati et al., 2024a).

VEGF and CCN1 are well known as important mediators of angiogenesis, and stimulate endothelial cell proliferation, migration, survival. Multiple co-stimulatory and positive feedback interactions exist between CCN1 and VEGFR2. CCN1 can bind to and activate VEGFR2 and downstream signaling pathways (including Akt) independently of VEGF and transcriptionally induce VEGFR2 and CCN1 expression in endothelial cells (Park et al., 2019). CCN1 also increases the expression of Src homology 2 domain-containing protein tyrosine phosphatase-1 (SHP-1) and its association with VEGFR2, which prevents endothelial cell hyperproliferation (Chintala et al., 2015). VEGF-stimulated angiogenesis depends on a crosstalk mechanism involving VEGFR2, αVβ3 integrin, and Syndecan-1 (Rapraeger et al., 2013). Our results are consistent with these earlier reports and confirm the role of VEGFR2 and Akt signaling in CCN1-induced angiogenesis, proliferation, and migration (Figure 2). MD-derived CCN1, a pro-angiogenic and anti-fibrotic regulator of VEGF signaling by crosslinking VEGFR2/ITGs/ECM, is strategically localized and an ideal physiological regulator to fine tune VEGF signaling efficiency in GEnCs.

CCN1 secretion by MD cells into the mesangium and glomerular tuft suggests that MD cells and their secreted molecules are important new players in the maintenance of a healthy glomerular endothelium, GEnCs, the glomerular filtration barrier (GFB), and in glomerular cell and matrix biology (Lausecker et al., 2022). Accordingly, CCN1 and/or other MD-derived factors were shown to regulate EPCs (Figures 1, 4), the number of GEnCs (Figures 1, 2) and podocytes (Gyarmati et al., 2024a), endothelial glycocalyx (Figure 3F), GFB albumin permeability (Figure 3D) and immune cell homing (Figure 3E). In addition to CCN1, other MD-derived secreted factors include PAPPA2, the #1 highest expressed MD-specific gene (Gyarmati et al., 2024a). MD-specific expression of PAPPA2 has been independently confirmed by at least two laboratories (Lindström et al., 2018; Cowley et al., 2016; Kumar et al., 2020). PAPPA2 is a known regulator of IGF bioavailability in the local tissue microenvironment by cleaving IGF binding proteins (IGFBPs) and releasing free IGF (Overgaard et al., 2001). CCN1’s IGFBP domain and IGFBP cleavage by PAPPA2 suggest potential for molecular interactions between CCN1 and PAPPA2 that need to be explored in future studies.

The present study addressed the human, disease, and therapeutic translational aspects of MD-derived CCN1. CCN1 protein was specifically expressed in MD cells in the human kidney, and its expression was reduced in lupus nephritis (LN) patients (Figures 1A, 3A) which is consistent with recently published results indicating significantly reduced CCN1 mRNA levels in LN and other CKD conditions (Gyarmati et al., 2024a). The specific changes in MD CCN1 expression have been reported for many types of CKD, but not for other MD cell markers like NOS1 and PTGS2 (Gyarmati et al., 2024a) indicating transcriptional regulatory mechanisms in MD cells in disease conditions. MD-derived biologicals had renoprotective effects in LN (Figure 3) similarly to what was demonstrated recently in a model of focal segmental glomerulosclerosis (Gyarmati et al., 2024a). The potent angiogenic effects of MD-derived factors was demonstrated directly and *in vivo* by the implantation of MD cells under the renal capsule (Figure 4). There are several benefits and potential bioengineering, biotechnology, and therapeutic applications of the presently demonstrated potent MD-regulated angiogenesis that could help address many major challenges in the CKD field. The potential applications of MD-regulated angiogenesis range from more efficient bioengineering of the vascular supply of biofabricated kidney tissues (Lim et al., 2022), to bioprinting, organ-on-chip technologies (Petrosyan et al., 2019), and generating recombinant MD-secreted proteins or MD cells for biologicals or cell-based therapies.

In summary, this study identified new regulatory mechanisms of renal angiogenesis by MD factors and validated their therapeutic potential for enhancing kidney tissue regeneration. The results of the present study may open the door to exploiting MD-targeting cell-based or biological approaches for tissue bioengineering and regenerative therapeutic developments.

## Data Availability

The raw data supporting the conclusions of this article will be made available by the authors, without undue reservation.
