# Supplementary figures and images for: Macula densa cell angiogenic mechanisms and their therapeutic potential in kidney disease
Source: Front Bioeng Biotechnol. 2025 Aug 6;13:1606230. doi: 10.3389/fbioe.2025.1606230 (PMC12365647; doi:10.3389/fbioe.2025.1606230)

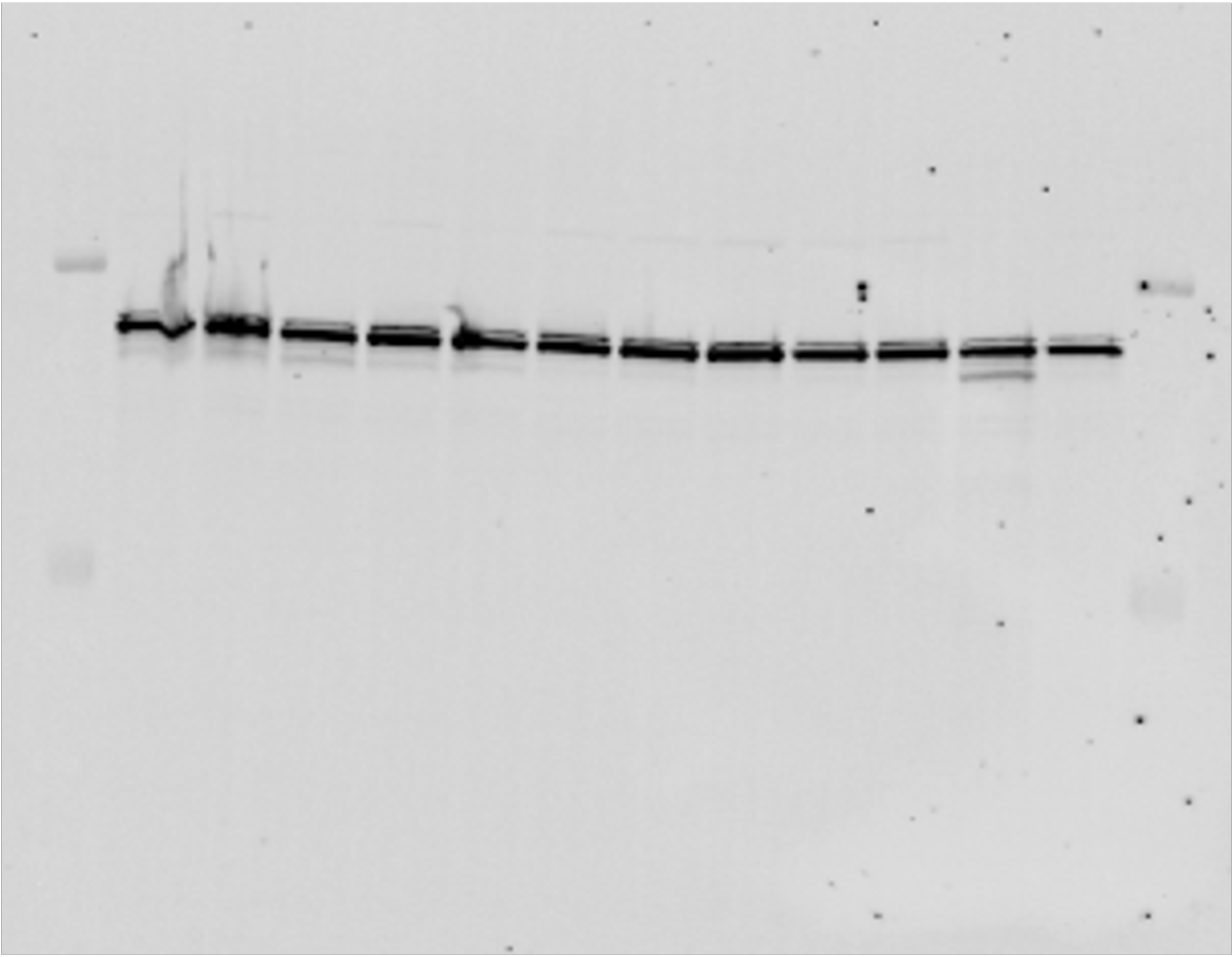

Supplement: Supplementary file 1 [file Image3.tiff]

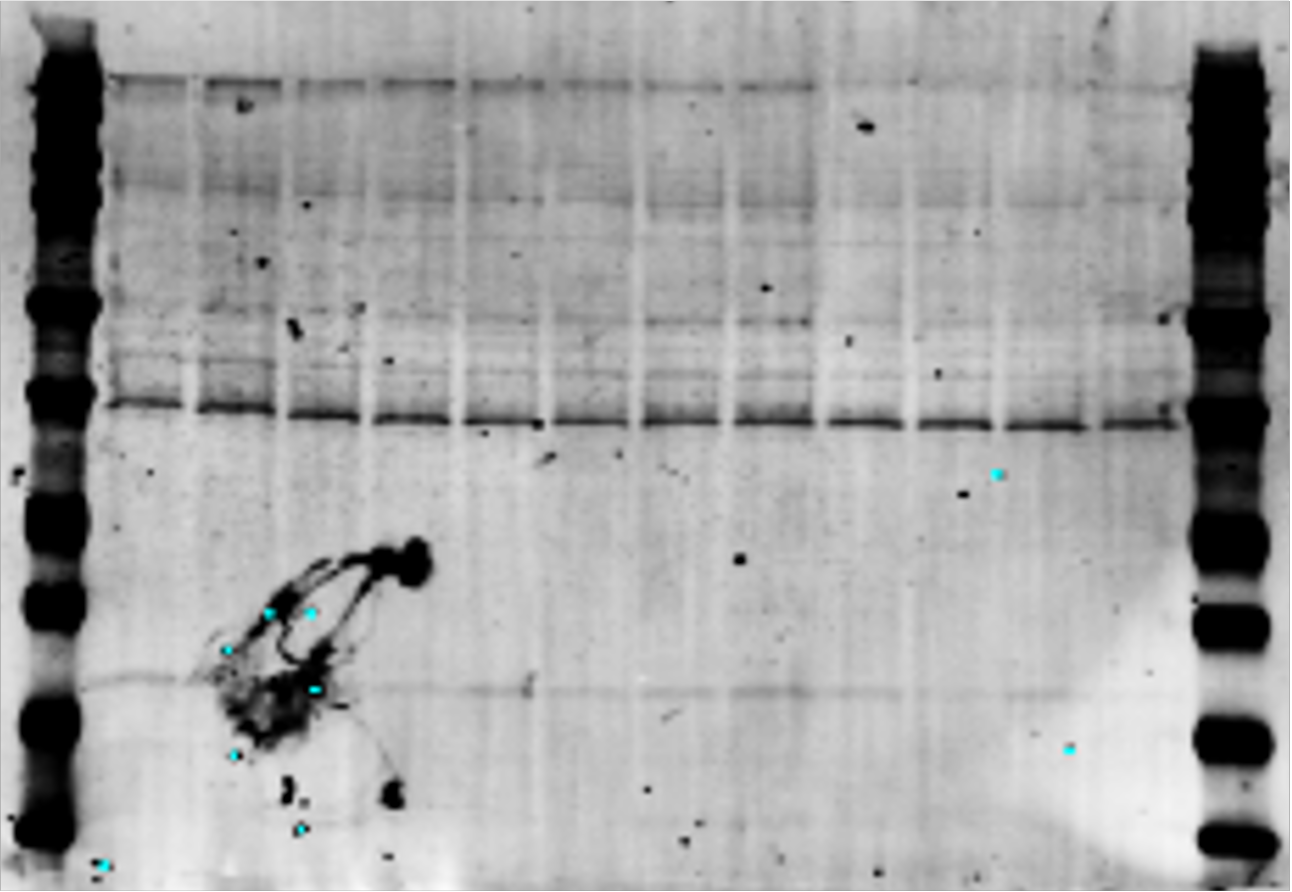

Supplement: Supplementary file 2 [file Image5.tiff]

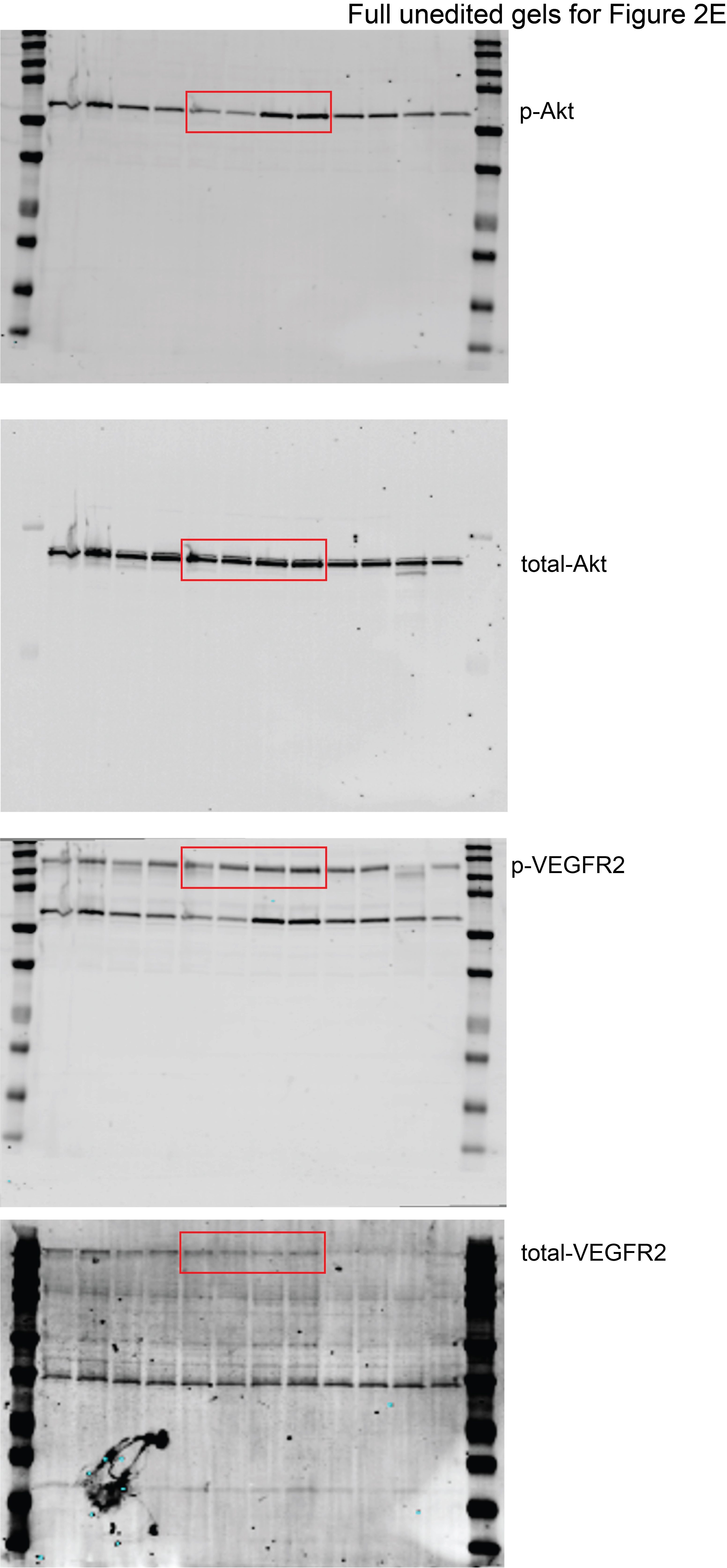

Supplement: Supplementary file 3 [file Image1.jpeg]

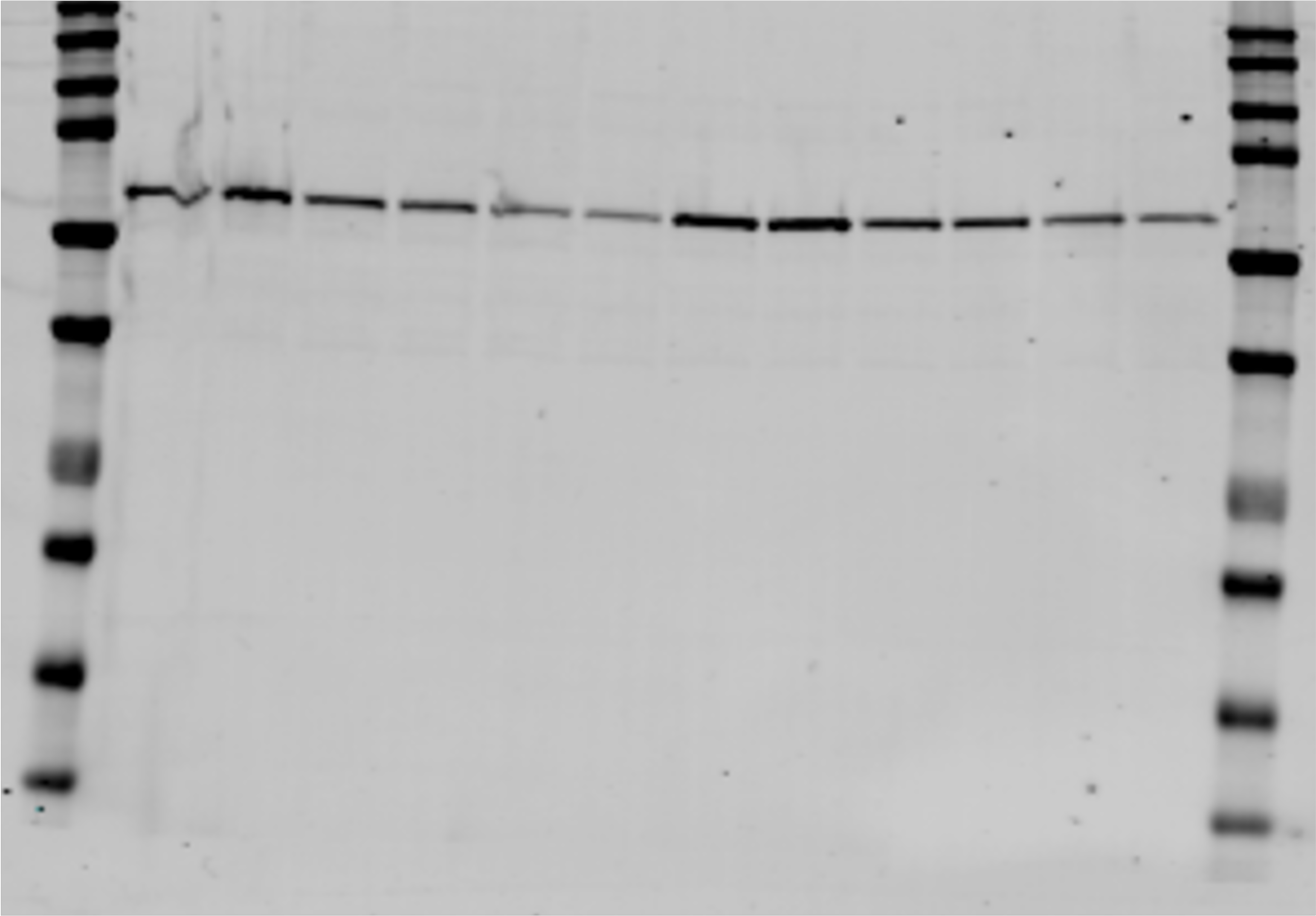

Supplement: Supplementary file 4 [file Image2.tiff]

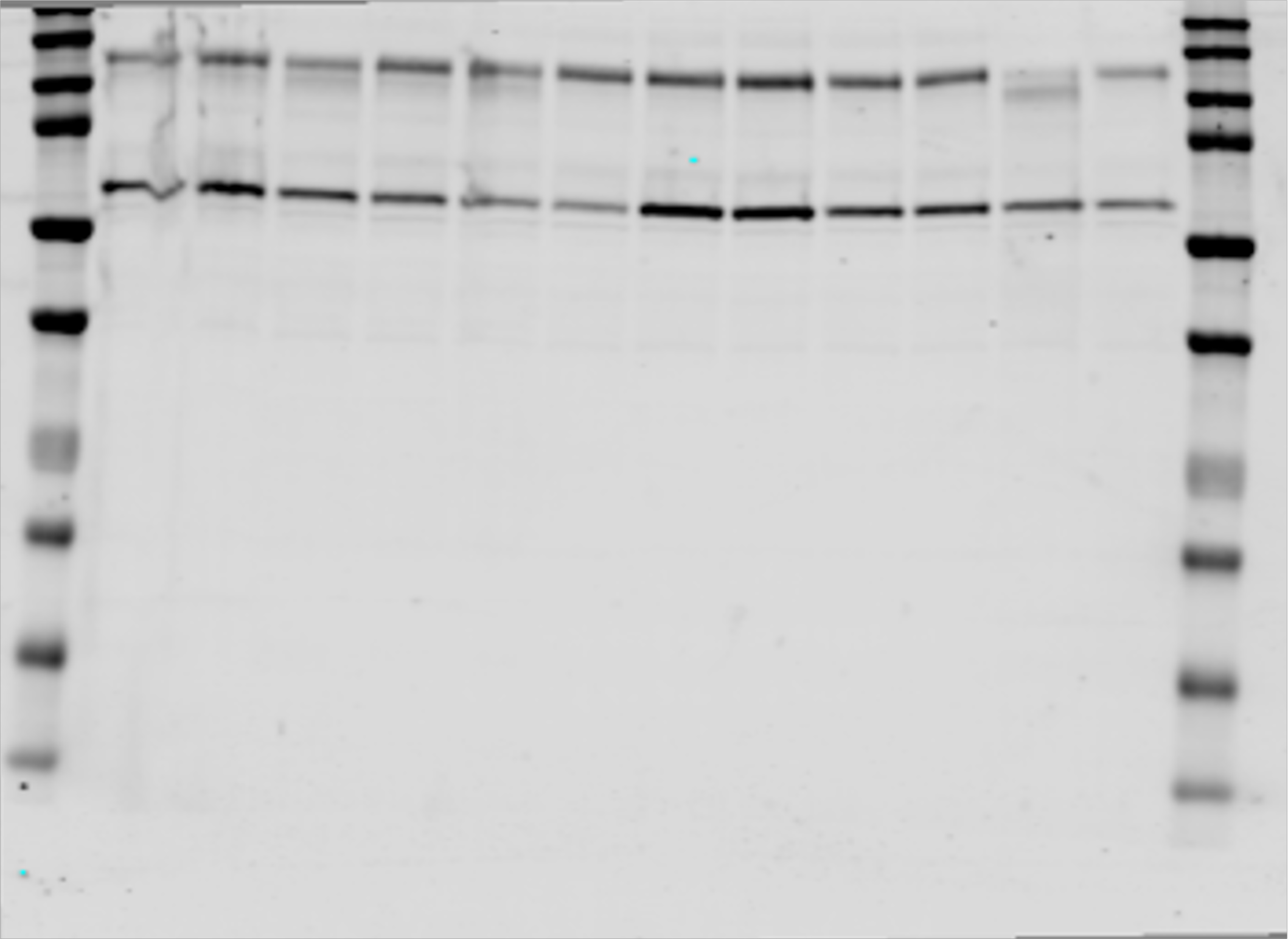

Supplement: Supplementary file 5 [file Image4.tiff]
